# Multi-Dimensional Assessment Approach to Assess Pesticide Manufacturing Industry Wastewater Toxicity

**DOI:** 10.3390/biology15090700

**Published:** 2026-04-29

**Authors:** Deling Fan, Jian Wang, Lili Shi, Lei Wang, Zheng Fang

**Affiliations:** 1 School of Biological and Pharmaceutical Engineering, Nanjing University of Technology, Nanjing 211816, China; fdl@nies.org; 2 Nanjing Institute of Environmental Science, Ministry of Ecology and Environment, Nanjing 210042, China; 18651888086@163.com (J.W.); sll@nies.org (L.S.)

**Keywords:** pesticide wastewater, non-target screening, neurodevelopmental toxicity, whole effluent toxicity, zebrafish embryos

## Abstract

Pesticide manufacturing industry wastewater is a complex mixture of potentially harmful components. If not properly treated, discharged effluents may pose serious risks to environment and organisms. In this study, influent and effluent wastewater samples from a pesticide factory were comprehensively non-screened by liquid chromatography high-resolution mass spectrometry, coupled with zebrafish embryo toxicity testing to assess whole effluent toxicity. A total of eight chemical groups were identified, including pesticides, antibiotics, nitrogen compounds, ketones, esters, amines and derivatives, other drugs, and other organic compounds. While wastewater treatment processes reduced most of the analyzed groups of compounds, compounds (e.g., 2-aminophenol, N-Nitrosodipropylamine, and carbamazepine) increased during the treatments. The influent samples were more toxic to zebrafish than the effluent samples in terms of lethality, teratogenic effects, developmental impacts, locomotor behavior, and neurotoxicity. The results showed that locomotor behavior was the most sensitive phenotypic toxicity endpoint, with significantly higher sensitivity than traditional acute lethal or teratogenic endpoints. Through a multi-dimensional assessment approach combining chemical screening, literature-based, risk ranking, and targeted quantification, we identified three predominant pesticide residues in the wastewater samples (both influents and effluents): hexaconazole, fenobucarb and isoprocarb. All three compounds exhibited additive or synergistic toxicity in zebrafish embryos. Exposure to ≥0.08% influent or ≥2% effluent increased inflammation (interleukin-1 beta, IL-1β), oxidative stress (copper/zinc superoxide dismutase, Cu/Zn-Sod), apoptosis (tumor protein p53, p53), and significantly impaired neurodevelopment in zebrafish larvae by altering the expression of sonic hedgehog a (shha), synapsin IIa (syn2a), and glial fibrillary acidic protein (gfap). This study suggests the necessity of incorporating non-apical endpoint (locomotor behavior) into whole effluent toxicity test, as this approach is essential for reducing the environmental risks posed by pesticide factory wastewater.

## Simple Summary

Pesticide factory wastewater contains a mixture of harmful substances, and improper discharge of such wastewater can pose severe threats to the environment and living organisms. Current regulatory standards such as GB 21523-2024, primarily rely on these apical markers (survival, reproduction and growth) but lack requirements for non-apical indicators (behavior, neurotoxicity). This study aimed to comprehensively analyze the pollutants and assess the toxicity of both untreated and treated wastewater from a pesticide plant, using chemical analysis and zebrafish embryo toxicity testing to identify potential risks. We found that changes in zebrafish locomotor activity served as a far more sensitive indicator of toxicity than traditional test endpoints focusing on lethality or birth defects. Exposing zebrafish to just 0.08% of influent or 2% of effluent triggered genetic changes. This study suggests that incorporating locomotor behavior assessments into full wastewater toxicity testing is essential, as this approach can more effectively reduce the environmental risks posed by pesticide manufacturing wastewater.

## 1. Introduction

China, as a major producer and consumer of pesticides, produced 2.498 million metric tons of pesticides in 2021, with over 100 million tons of wastewater discharged annually [1]. However, Conventional wastewater treatment processes often cannot effectively remove all pollutants from pesticide factory effluent [2]. Some contaminants can be transformed during wastewater treatment, and the transformation products can be more toxic or more resistant to degradation than the parent compounds, meaning that the treatment increases the environmental risks [3]. Pesticides frequently enter groundwater and surface water systems through leaching and runoff, posing significant threats to aquatic organisms and potentially infiltrating the human food chain, thereby exerting adverse effects on human health [4]. While extensive research has addressed the toxicological impacts of pesticide residues [5,6], current regulatory frameworks—such as GB 21523-2024 [7]—remain focused on traditional apical markers. For instance, the standard primarily stipulates a minimum 90% survival rate for zebrafish (*Danio rerio*) embryos at a 6-fold dilution, failing to account for more sensitive non-apical endpoints. These traditional endpoints lack the sensitivity to detect non-apical impairments, such as functional impairments at the molecular or behavioral levels. It is increasingly evident that wastewater effluent can have effects on fitness-related biological functions, and that non-apical endpoint (behavior, neurotoxicity) should be considered [8].

Comprehensive assessment of the environmental impacts of pesticide plant effluent discharge requires that the contaminants be accurately identified and quantified, and their toxicities systematically assessed. The zebrafish (*Danio rerio*) is a well-established aquatic model organism with a high degree of genetic homology to humans [9,10,11]. Species Sensitivity Distribution (SSD) model statistically aggregates diverse reported toxicity data to quantify the distribution of species sensitivities, enabling estimation of hazardous concentrations and the derivation of Predicted No-Effect Concentration (PNEC) [9]. Therefore, we integrated non-target chemical screening with the assessment of both apical and non-apical endpoints, enabling a comprehensive evaluation of pesticide wastewater toxicity.

The objective of this study was to comprehensively analyze influent and effluent water samples from a pesticide plant wastewater treatment facility using non-target screening and zebrafish embryo toxicity tests. Chemical analysis was performed using liquid chromatography (LC) high-resolution mass spectrometry (HRMS). We evaluated the impact of influent and effluent samples on zebrafish embryonic development, locomotor behavior, and neurotoxicity, alongside associated gene expression profiles. Ultimately, we utilized both the SSD approach and the assessment factor (AF) method to compare and derive PNEC values, thereby establishing robust protective thresholds. These results are expected to contribute to the development of effective pollution control and environmental protection strategies.

## 2. Materials and Methods

### 2.1. Reagents

Reverse transcription kits and SYBR Green assay kits were purchased from Vazyme Biotech (Nanjing, China). Eighty-three analytical standards (purity ≥ 99.0%) were purchased from Alta Scientific (Tianjin, China) and stored at −18 °C in the dark for up to 6 months, and the detailed information is provided in Table S2. High-performance-LC grade methanol and acetonitrile were purchased from Merck KGaA (Darmstadt, Germany). Ultrapure water (Type I, ISO 3696:1987, 18.2 MΩ·cm) was prepared by a Milli-Q purification system (Millipore, Burlington, MA, USA). Oasis HLB solid phase cartridges (6 cc, 500 mg) were purchased from Waters (Milford, MA, USA). Analytical standards of isoprocarb (MIPC, CAS No. 2631-40-5), hexaconazole (HEX, CAS No. 79983-71-4), and fenobucarb (BPMC, CAS No. 3766-81-2) were purchased from Alta Scientific Co., Ltd. (China) with a purity of ≥99.0%. Stock solutions were prepared in acetone and stored at −20 °C in the dark before use.

### 2.2. Instrument

Whatman glass fiber filters (GF/F, pore size 0.7 µm) were purchased from GE Healthcare Bio-Sciences (Pittsburgh, PA, USA). A Visiprep SPE vacuum manifold was purchased from Sigma-Aldrich (St. Louis, MO, USA). Samples were analyzed using an ultra-performance LC Q Exactive Focus Orbitrap HRMS instrument (Thermo Fisher Scientific, Waltham, MA, USA).

### 2.3. Zebrafish Maintenance and Embryo Collection

Zebrafish (common wild-type AB zebrafish, motor-neuron-specific transgenic zebrafish Tg (hb9:eGFP), and central-nervous-system-specific transgenic zebrafish Tg (huc:eGFP)) were provided by the Institute of Hydrobiology, Chinese Academy of Science. The zebrafish were maintained in aerated tap water at 27.5–28.5 °C and pH 6.5–7.5 with a 14 h light and 10 h dark cycle [12].

In the evening before the start of an experiment, male and female zebrafish at a ratio of 1:2 were placed in partitioned breeding boxes. In the following day, the partition was removed, and illumination was applied, allowing the male and female zebrafish to chase each other and spawn. Fertilized eggs were collected, and healthy fertilized eggs identified under a microscope were selected for the exposure experiments. Following the WET framework, we assessed the aggregate effects of the complex pesticide effluent using zebrafish embryos as a standardized vertebrate model. A multi-concentration dilution series was employed to characterize the toxicity profile and establish dose–response relationships. The animal experiments were performed in accordance with the Experimental Animal Care and Use Guidelines of the Nanjing Institute of Environmental Sciences (IACUC-20220319).

### 2.4. Collection and Pre-Treatment of Water Samples

In August, September, and November 2022, 2 L samples of influent and effluent wastewater were collected by grab sampling from the wastewater treatment plant at a pesticide plant (East China), respectively. These individual samples were pooled to form a single composite sample for final chemical and toxicological analysis. The treatment scheme comprises physicochemical pretreatment (e.g., Fenton-like oxidation), followed by a biological system (A/O process) and final advanced oxidation for polishing. Each water sample was adjusted to pH 2–4 by adding hydrochloric acid and then passed through a glass fiber filter with 0.7 μm pores. A 500 mL aliquot of the filtered water was then passed through an Oasis HLB 6 mL solid-phase extraction cartridge that had been activated with methanol. The loading flow rate was ~5 mL/min. The loaded HLB cartridge was rinsed with 10 mL of ultrapure water and dried under vacuum for 30 min under negative pressure. The analytes were then eluted with 10.0 mL of methanol, and the eluate was evaporated to dryness under a stream of nitrogen and then reconstituted in 1 mL of methanol. The extract was vortexed for 1 min and analyzed by LC-HRMS.

### 2.5. Detection and Analysis of Chemicals in the Water Sample Extracts

Chromatography was performed using a Hypersil Gold C18 column (50 mm long, 2.1 mm i.d., 1.9 µm particle diameter, Thermo Fisher Scientific, Waltham, MA, USA). The column was kept at 40 °C. The flow rate was 0.3 mL/min, and the injection volume was 5 µL. For positive ion mode, mobile phase A was 0.1% formic acid in water, and mobile phase D was acetonitrile. For negative ion mode, mobile phase A was 0.05% ammonia solution, and mobile phase D was acetonitrile. The gradient elution program was: 0 min, 90% A: 10% D; 0–2 min, 90% A: 10% D; 2–3 min, 70% A: 30% D; 3–6 min, 50% A: 50% D; 6–9 min, 40% A: 60% D; 9–15 min, 0% A: 100% D; 15–23 min, 0% A: 100% D; 23–23.5 min, 90% A: 10% D; and 23.5–25 min, 90% A: 10% D.

Ionization was performed using an electrospray ion source with a source temperature of 375 °C and a spray voltage of 3500 V. The sheath gas flow rate was 40 arbitrary units (arb), the auxiliary gas flow rate was 10 arb, and the counter gas flow rate was 0 arb. The S-lens RF value was 50. The auxiliary gas pressure was 10 arb, and nitrogen was used as both spray gas and collision gas. Full MS/ddMS2 + Discovery scan mode was used, with samples introduced in both positive and negative ion modes. Full scan resolution was 70,000 for the first level, and the data-dependent scan resolution was 17,500 for the second level. The scan range was 100–1500 *m*/*z* [13]. The automatic gain control for the first-level scan was 1.0 × 10^6^, and the ion injection time was 100 ms. The automatic gain control for the data-dependent second-level scan was 1.0 × 10^5^, the maximum ion injection time was 100 ms, the isolation window was 3.0 *m*/*z*, the collision energy was 40 eV, the loop count was 3, and the dynamic exclusion time was 10.0 s.

### 2.6. Zebrafish Toxicity and General Developmental Toxicity Tests

The test was conducted following the OECD TG 236 guideline [14]. Preliminary acute toxicity assays were performed at 1%, 10%, and 100% (*v*/*v*) to define the effective concentration range. The results revealed that influent samples exhibited no acute toxicity at dilutions > 10%, whereas effluent samples showed no acute toxicity at dilutions > 50%. Consequently, the test dilution ranges were set to 0.08–10% for influent and 0.4–50% for effluent to ensure environmentally realistic exposure and capture potential toxic responses. After preliminary experiments, the influent water samples were diluted to concentrations of 0.08%, 0.4%, 2%, and 10%, the effluent water samples were diluted to concentrations of 0.4%, 2%, 10%, and 50% with aerated water, and zebrafish embryos were exposed to these diluted samples. The control was blank aerated water. Healthy zebrafish embryos (<3 hpf) were individually exposed in 24-well plates (*n* = 24 per group, 3 replicates) and maintained at 26 °C ± 1 °C under a 14:10 h light/dark cycle. The test met all validity criteria, with control mortality remaining below 10%. In the acute exposure experiment, healthy fertilized zebrafish eggs from <3 h post-fertilization (hpf) through to the larval stage at 144 hpf were exposed to the test solutions [15]. Half of the test solution was replaced each day to keep the concentration stable. Developmental toxicity parameters, including mortality, hatching rate, heart rate, and body length, were recorded at 24, 48, 72, 96, and 144 hpf using a SMZ25 stereomicroscope with the ability to measure fluorescence (Nikon, Tokyo, Japan). To further evaluate the contribution of specific contaminants to the observed toxicity, individual acute toxicity tests were performed using analytical standards of HEX, BPMC, and MIPC. Zebrafish embryos (<3 hpf) were exposed to a series of concentrations. The experimental conditions (temperature, light cycle, and pH) were identical to those described for the wastewater testing. The concentrations for the individual and mixture validation tests were strictly determined based on the quantitative analysis of the effluent sample. Specifically, zebrafish embryos were exposed to 0.337 µg/L of MIPC, 2.076 µg/L of HEX, and 3.225 µg/L of BPMC separately to assess their individual toxicity. To evaluate the combined effects, a mixture group was prepared by combining all three compounds at their respective effluent concentrations. For the determination of dose–response relationships, a geometric series of five concentrations (with a dilution factor of 2.0) was employed, ranging from 1 to 20 µg/L.

### 2.7. Locomotor Behavioral Analysis

At 144 hpf, twenty-four zebrafish embryos were randomly selected from each exposure group and individually placed in wells of a 24-well plate for behavioral testing, as described in a previous publication [14]. EthoVision XT 18 (Noldus Information Technology, Wageningen, The Netherlands) was used to record and analyze the locomotor trajectories of zebrafish larvae. The distance traveled, dwell time, and movement speed were recorded for the zebrafish larvae in each exposure group. Movement trajectory maps and thermograms were produced and used to assess the locomotor abilities of the zebrafish larvae.

### 2.8. Evaluation of Neurodevelopmental Toxicity in Zebrafish

Transgenic zebrafish Tg (hb9:eGFP) motor neuron labeled embryos and zebrafish Tg (huc:eGFP) central nervous system labeled embryos were exposed to different concentrations of influent and effluent samples consecutively for 72 and 144 hpf [12]. Fifteen zebrafish larvae were randomly selected from each exposure group. Fluorescence images of the central nervous system regions of these zebrafish larvae were captured using a Nikon SMZ25 fluorescence microscope. The fluorescence intensities in the images were determined using NIS-Elements D 4.60 software, and the results were statistically analyzed.

### 2.9. Real-Time PCR

Total RNA was isolated from zebrafish larvae from the different exposure groups using TRIzol [16]. The isolated RNA was then reverse transcribed into cDNA using 5× NO RT Control Mix, 5× Hiscript qRT Super Mix, RNase-free ddH2O, and 4× Gdna Wiper Mix. The reverse transcription product was then mixed with 2× ChamQ Universal SYBR qPCR premix and appropriate ratios of target gene-specific primers. Primer sequences for genes associated with inflammation, oxidative stress, apoptosis, and neurodevelopment are shown in Supplementary Table S1. Measurements were performed using a fluorescent quantitative PCR instrument. The relative expression level for each gene was calculated using the 2^−ΔΔCT^ method, and the results were normalized to β-actin.

### 2.10. Data Analysis

SSD was used to derive the water quality criteria of compound, with calculations performed via the National Software for Environmental Quality Criteria-SSD (EEC-SSD) V1.0 [17]. Based on the worst-case scenario according to the Guidance on Information Requirements and Chemical Safety Assessment by the European Chemicals Agency (2016) [18], the PNECs are calculated from the minimum half-maximal concentration (EC_50_ or LC_50_) and assessment factor (AF, typically 1000 for acute toxicity). The equations are as follows:

(1) $${HC}_{5}=exp(\mu +z_{0.05}\times \sigma )$$

(2) $$PNEC=\frac{{HC}_{5}}{AF}$$

(3) $$RQ=\frac{MEC}{PNEC}=\frac{MEC}{E(L)C_{50}}\times AF$$

(4) $${\mathrm{TEF}}_{i}=\frac{{\mathrm{PNEC}}_{A}}{{\mathrm{PNEC}}_{i}}$$

(5) $$\sum (C_{\mathrm{effluent},i}\times {\mathrm{TEF}}_{i})\leq {\mathrm{PNEC}}_{A}\times \frac{{\mathrm{DF}}_{1}\times {\mathrm{DF}}_{2}}{1-R_{\mathrm{plant}}}$$

where μ and σ represent the mean and standard deviation of the log-transformed toxicity values (e.g., EC_50_, LC_50_), and z_0.05_ (−1.645) denotes the critical value from the standard normal distribution at the lower 5th percentile. This value was obtained from the CDFs of the standard normal distribution (mean = 0, σ = 1), where the probability of observing a value below −1.645 is 0.05. When applied in SSD modeling, this threshold was illustrated graphically in the SSD curve as the point at which 5% of the most sensitive species were expected to be affected, providing a conservative hazard benchmark for ecological risk assessment and supporting the derivation of protective regulatory limits. Local water quality criteria is water quality criteria for aquatic life, with unit of μg/Lor mg/L. HC_5_ is hazardous concentration for 5% of species, calculated by the national eco-environmental benchmark calculation software-SSD method. PNEC is long-term water quality criteria for aquatic organisms, and MEC refer to the measured environmental concentrations. AF is Assessment Factor. The value of the AF was determined comprehensively based on the quantity of data used to derive the criteria, the taxonomic coverage of tested species, and the goodness-of-fit of the SSD, among other factors. It generally ranges from 2 to 3, which is aligned with the Technical Guideline for Deriving Water Quality Criteria for Freshwater Organisms (HJ 831—2022). Specifically, an AF of 2 was applied when the number of species with valid toxicity data (*n*) exceeded 15. For datasets where *n* ≤ 15, an AF of 3 was generally adopted to ensure a sufficient margin of safety. One-way analysis of variance was used to analyze the differences between the treatments, which were performed using SPSS (version 16.0; SPSS, Inc., Chicago, IL, USA). *C*_effluent_ is the concentration of industrial effluent concentration, Rplant is the degradation rate in the wastewater treatment plants (WWTP), the respective dilution factors for the effluent from the enterprise outfall into WWTP in chemical industrial park (DF_2_), and the dilution factors for effluent WWTP entering surface water (DF_1_), with a default value of 36.1 [19]. Furthermore, the Toxic Equivalency Factor was utilized to evaluate the cumulative risk of the identified chemical mixtures.

## 3. Results

### 3.1. Identification of Contaminants in the Influent and Effluent Samples

Non-targeted LC-HRMS was used to screen and quantify the pollutants in the influent and effluent samples from the pesticide wastewater treatment plant (Table S2). As shown in Table 1, the total pollutant concentrations in the influent and effluent were 73.22 and 69.20 μg/L, respectively. More chemicals were detected in the effluent (62 species) than in the influent (59 species), mainly because more nitrogenous compound species were found in the effluent than in the influent.

**Figure 1:**
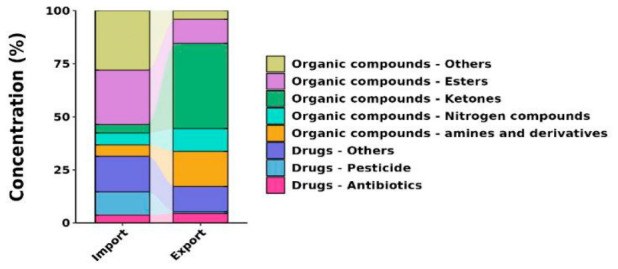
Concentrations and composition ratios of chemicals in the influent and effluent samples.

Stacked plots of the pollutant concentrations and compositions indicated that the pesticide, pharmaceutical (except antibiotic), ester, and other organic compound concentrations were lower in the effluent than the influent and that the removal efficiencies were 93%, 34%, 58%, and 86%, respectively (Figure 1). However, nitrogenous compounds, ketones, amines, and derivatives were found at higher concentrations and composition ratios in the treated effluent than in the influent, with the concentrations of all nitrogenous compounds increasing (and four nitrogenous compounds being detected in the effluent but not the influent).

### 3.2. General Developmental Toxicity of the Influent and Effluent Water Samples to Zebrafish Embryos

**Figure 2:**
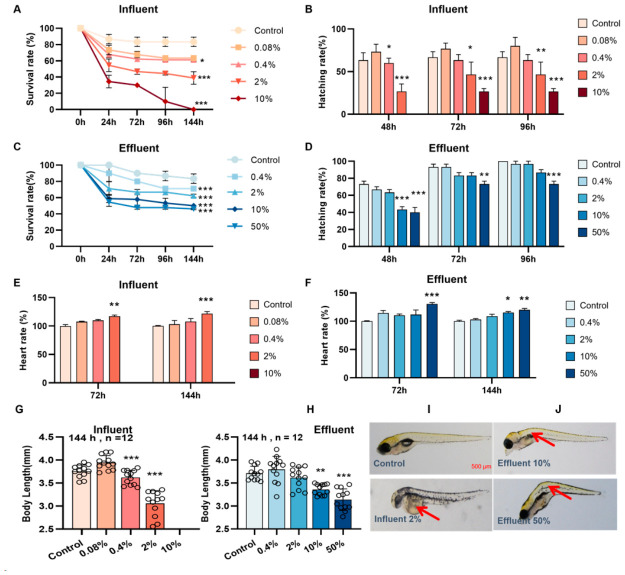
General developmental toxicity. (**A**,**C**) Survival rate, (**B**,**D**) hatching rate, (**E**,**F**) heart rate, (**G**,**H**) body length, and (**I**,**J**) Representative images of zebrafish at 144 hpf. Data were analyzed using one-way ANOVA, * *p* < 0.05, ** *p* < 0.0 1, *** *p* < 0.001.

As shown in Figure 2A,C, at 144 hpf, larval survival was significantly lower (*p* < 0.05) for zebrafish embryos exposed to the various influent and effluent sample concentrations than for the control group. All zebrafish larvae at 144 hpf died, when the influent concentration was 10% or higher (Figure 2A). In contrast, 45–50% of zebrafish larvae survived at 144 hpf at effluent concentrations of 10% and 50% (Figure 2C). The zebrafish larva hatch abilities at 48, 72, and 96 hpf were significantly lower (*p* < 0.05) for the 2% and 10% influent groups than the control group (Figure 2B). The zebrafish larva hatch abilities were significantly lower (*p* < 0.05) for the 10% effluent group at 48 h and the 50% effluent group at 48, 72, and 96 h than the control group (Figure 2D). At 72 hpf, the zebrafish heart rate was significantly higher (*p* < 0.05) for the 2% influent group and 50% effluent group than for the control group. At 144 hpf, the zebrafish heart rate was significantly higher (*p* < 0.05) for the 2% influent group and the 10% and 50% effluent groups than for the control group (Figure 2E,F). Pictures of typical zebrafish in each test group at 144 hpf are shown, with malformations such as pericardial edema and spinal curvature found in the 2% influent group and the 10% and 50% effluent groups. Statistical analysis indicated that exposure to >0.4% influent or >10% effluent resulted in statistically significantly decreased body length (Figure 2G,H). These results indicated that the influent at a concentration > 0.4% and the effluent at a concentration > 10% caused developmental impairments in zebrafish larvae (including decreased survival and hatchability, increased heart rate, decreased body length, and malformations such as pericardial edema and spinal curvature) and that more developmental toxicity was caused by the influent than the effluent.

### 3.3. Effects of the Influent and Effluent Samples on Zebrafish Locomotor Behavior

**Figure 3:**
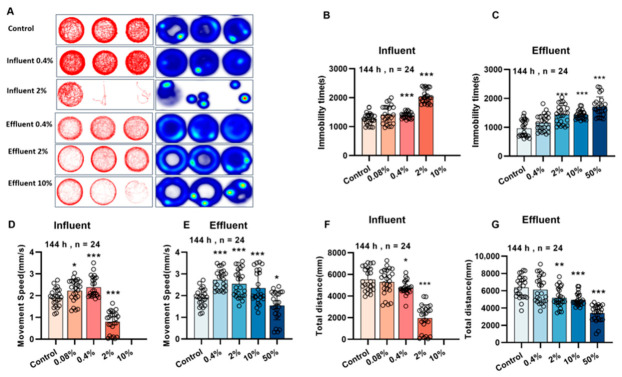
Abnormalities in zebrafish locomotor behavior. (**A**) Behavioral trajectories 4. (**B**,**C**) Dull time, (**D**,**E**) movement speed, and (**F**,**G**) total distance traveled at 144 hpf, *n* = 24. Data were analyzed using one-way ANOVA, * *p* < 0.05, ** *p* < 0.01, *** *p* < 0.001.

The behavioral analysis results are shown in Figure 3. The dull time was significantly higher (*p* < 0.05), and the total travel distance was significantly lower (*p* < 0.05) for zebrafish larvae in the influent (0.4% and 2%) and effluent (0.4%, 2%, and 10%) groups than the control group (Figure 3B,C,F,G). The movement speed of zebrafish larvae was significantly different (*p* < 0.05) from the control group in the influent (0.08–10%) and effluent (0.4–50%) groups, showing an initial increase followed by a decrease as the influent or effluent concentration increased (Figure 3D,E). These results indicated that an influent sample concentration > 0.08% or an effluent sample concentration > 0.4% caused abnormal locomotor behavior, including increased lag time, decreased total travel distance, and altered locomotor speed. The locomotor behavior was affected more by the influent than the effluent.

### 3.4. Developmental Toxicity of the Influent and Effluent to Zebrafish Motor Neurons

**Figure 4:**
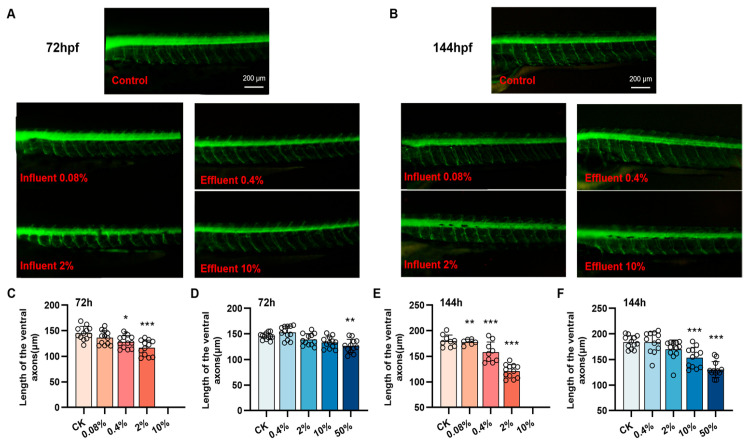
Motor neuron developmental toxicity. Representative images of Tg (hb9:eGFP) zebrafish at (**A**) 72 hpf and (**B**) 144 hpf. Ventral axon lengths for Tg (hb9:eGFP) zebrafish at (**C**,**D**) 72 hpf and (**E**,**F**) 144 hpf, *n* = 12. Data were analyzed using one-way ANOVA, * *p* < 0.05, ** *p* < 0.01, *** *p* < 0.001.

Two transgenic zebrafish strains (hb9-eGFP and huc-eGFP) were used to visualize the toxicities of the influent and effluent to the zebrafish nervous system. As shown in Figure 4, at 72 hpf, the motor neuron axon length was significantly lower (*p* < 0.05) for Tg (hb9:eGFP) zebrafish exposed to 0.4% or 2% influent or 50% effluent than for the control group (Figure 4A,C,D). At 144 hpf, the motor neuron axon length was significantly lower (*p* < 0.05) for zebrafish exposed to ≥0.08% influent or ≥10% effluent than for the control group (Figure 4B,E,F). These results indicated that ≥0.08% influent or ≥10% effluent caused motor neuron axon shortening in zebrafish larvae and that the influent was more toxic than the effluent.

### 3.5. Developmental Toxicity of the Influent and Effluent to the Zebrafish Central Nervous System

**Figure 5:**
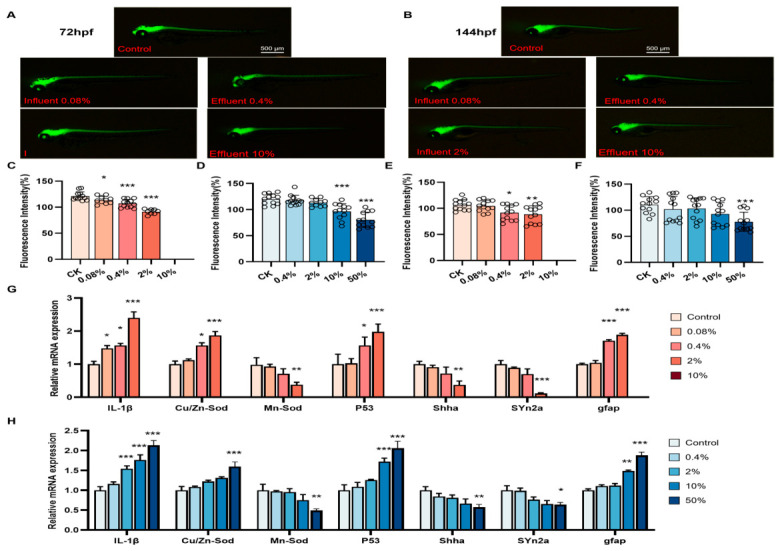
Central nervous system developmental toxicity. Representative images of Tg (huc:eGFP) zebrafish at (**A**) 72 hpf and (**B**) 144 hpf. Central nervous fluorescence intensity of Tg (huc:eGFP) zebrafish at (**C**,**D**) 72 hpf and (**E**,**F**) 144 hpf, *n* = 12. (**G**,**H**) Expression of genes related to inflammation (IL-1β), oxidative stress (Cu/Zn-Sod and Mn-Sod), apoptosis (p53), and neurodevelopment (Shha, Syn2a, and gfap). Data were analyzed using one-way ANOVA, * *p* < 0.05, ** *p* < 0.01, *** *p* < 0.001.

Tg (huc:eGFP) zebrafish embryos were exposed to various influent and effluent concentrations. At 72 hpf, zebrafish exposed to >0.08% influent or >10% effluent had significantly lower (*p* < 0.05) central nervous fluorescence intensities than the controls (Figure 5A,C,D). At 144 hpf, the central nervous system fluorescence intensity was significantly lower (*p* < 0.05) for the ≥2% influent and ≥50% effluent groups than the control group (Figure 5B,E,F). These results indicated that the influent at a concentration > 0.08% and the effluent at a concentration > 10% caused central nervous system damage in zebrafish larvae, and that the influent was more toxic than the effluent.

The toxicity of the influent and effluent to the zebrafish nervous system was investigated further by investigating mRNA expression of key genes involved in inflammation, oxidative stress, apoptosis, and neurodevelopment by PCR (Figure 5G,H). The results indicated that significantly more (*p* < 0.05) inflammatory factor (IL-1β) expression occurred in the >0.08% influent groups and >2% effluent groups than the control group. Exposure to >0.4% influent or >10% effluent significantly (*p* < 0.05) changed expression of genes related to oxidative stress (Cu/Zn-Sod and Mn-Sod) and apoptosis (p53). Expression of key genes for neurodevelopment (Shha, Syn2a, and gfap) was significantly (*p* < 0.05) changed by exposure to >2% influent or >10% effluent. These results indicated that exposure to >0.08% influent or >2% effluent increased inflammation, oxidative stress, and apoptosis in zebrafish larvae and significantly affected neurodevelopment, and that the influent was more toxic in terms of neurodevelopment than the effluent.

### 3.6. Toxicity Match of Detected Substances and Environmental Water Samples

In general, additive or synergistic toxicity exhibited a significant effect on acute toxicity (2% effluent groups), hatching rate (10% effluent groups), body length (10% effluent groups), heart rate (10% effluent groups), locomotor behavior (0.4% effluent groups), neurodevelopment movement (10% effluent groups), central neurodevelopment (2% effluent groups), and genotoxicity (>2% effluent increased inflammation, oxidative stress, and apoptosis). The locomotor behavior is among the most sensitive indicators for detecting wastewater toxicity, with significantly higher sensitivity than traditional acute lethal or teratogenic endpoints.

A risk ranking based on the ratio of the detected concentration to the acute toxicity value in fish revealed that MIPC, HEX, and BPMC were the most significant three chemicals (Table 2). However, due to the scarcity of chronic toxicity data (NOEC values) for certain detected compounds, the calculation of C/NOEC ratios was not feasible for all substances. To validate the findings, we purchased authentic standards of these three substances for verification and quantitative analysis. The results indicated that these three chemicals exhibited significantly increased concentrations in the influent and effluent samples. We conducted acute toxicity experiments with zebrafish exposed to BPMC, HEX, and MIPC individually and in a mixture at concentrations detected in the effluent sample. The results indicated that individual exposure to these substances did not lead to mortality in zebrafish embryos, whereas the mixed exposure group for BPMC, HEX, and MIPC exhibited a significantly lower body length, movement speed, and movement distance, compared to the control (Figure S1). Toxic effects caused by other factors have been excluded due to the similarity of water quality indicators (pH, dissolved oxygen, and others) between simulated wastewater and actual wastewater. The toxicity validation experiments demonstrated that the mixed exposure of these three substances at environmental concentrations exhibited toxic effects similar to those of the effluent sample, with additive or synergistic toxicity observed in the mixed exposure.

The SSD-HC_5_ of HEX was determined to be 0.925 mg/L. Using an assessment factor (AF) of 3, we calculated that the acute toxicity local water quality criteria for HEX was 0.185 mg/L. The HEX concentration was measured at 3.166 μg/L in influent and 2.076 μg/L in effluent, representing a removal efficiency of 34.4% during wastewater treatment. To assess the ecotoxicological potential of HEX residues in aquatic environments, we systematically reviewed existing literature through PubChem, Web of Science, and Scopus databases using the keywords “zebrafish” and “hexaconazole.” As a broad-spectrum triazole fungicide, HEX is extensively utilized in global agricultural practices to improve crop yields. Lee et al. demonstrated that HEX exposure triggers apoptotic cell death, DNA fragmentation, and inflammatory responses during zebrafish embryogenesis. Transgenic zebrafish models (olig2:dsRed, fli1:eGFP, and l-fabp:dsRed) revealed compound-specific organotoxicity, manifesting as neurodevelopmental abnormalities, cardiovascular dysfunction, and hepatic damage, respectively [20]. The toxicity data were collected from ECOTOX (https://cfpub.epa.gov/ecotox/) (Table S3). For BPMC, the SSD-HC_5_ value was determined to be 0.07 mg/L, with a water quality criterion of 0.014 mg/L (applying an assessment factor of 5). MIPC showed an HC_5_ of 2.233 mg/L, with the water quality criterion of 0.446 mg/L. Environmental monitoring detected these compounds in effluent samples at concentrations of 3.225 μg/L (BPMC) and 0.337 μg/L (MIPC). Zhu et al. revealed that BPMC induced developmental neurotoxicity in zebrafish characterized by motility reduction, motor neuron damage, axon and myelin degeneration [21]. Wang et al. explored MIPC causes neurotoxicity of zebrafish embryos through oxidative stress-induced apoptosis [22]. The results suggest that the toxic effects of BPMC, HEX, and MIPC exposure align with the toxic effects observed in the effluent sample, particularly regarding behavioral abnormalities identified as the most sensitive toxic endpoint. Tables S4 and S5 demonstrate that while the chemical concentrations in the industrial effluent were well within the chemical industrial park discharge limits derived from both SSD and AF methods, the 10% of effluent still exceeded the PNEC_AF_ threshold. This exceedance is highly consistent with the significant neurotoxicity observed in our zebrafish assays. Although the concentrations of these principal pollutants were below their respective PNEC values or regulatory discharge limits, the potential presence of unidentified contaminants, transformation products, or joint toxic effects in the wastewater cannot be ignored. This highlights the critical need to integrate effect-based biological monitoring into current environmental assessment frameworks to safeguard aquatic ecosystems from complex chemical mixtures.

## 4. Discussion

Accelerating global industrialization has made pesticide production an increasing important industry for ensuring reliable agricultural yields. However, the large amounts of wastewater produced by pesticide factories contain various pollutants that pose serious risks to the environment [23]. Addressing this problem has therefore become an urgent priority [24]. Accurately identifying pollutants in wastewater and determining the environmental fates and ecotoxicological behaviors of the pollutants are essential to develop effective strategies for preventing pollution and protecting the environment [25]. Similar with previous studies, we detected various pollutants, including pesticides, pharmaceuticals, and various other organic chemicals, in the influent and effluent samples [26]. Regarding wastewater management, conventional WWTPs are capable of effectively removing certain emerging organic micropollutants, although they were not originally designed to eliminate these compounds at such low concentrations [19]. However, if treatment is inadequate, these contaminants can enter and accumulate in the human body through various exposure pathways, including inhalation and the ingestion of contaminated drinking water and food [13,27]. Among the pollutants identified in this study, several have well-documented pathological effects. For instance, HEX has been demonstrated to exert endocrine-disrupting effects [28], with previous reports showing that HEX can decrease the activity of aromatase (CYP19A1) in human adrenocortical carcinoma cells [29]. Furthermore, naphthalen-2-amine is a confirmed IARC Group 1 human carcinogen, strongly linked to the development of bladder cancer [30]. Similarly, the plasticizer diethyl phthalate is a known endocrine disruptor associated with reproductive dysfunction and insulin resistance [31]. Particularly, children are more susceptible to the short-, medium-, and long-term effects of these pollutants, especially pesticides, due to their lower body mass, higher metabolic rates, and ongoing physiological development [32]. The continuous discharge and accumulation of these pollutants could pose severe threats to both aquatic ecosystems and human health [33]. While the pathological pathways of some identified pollutants are well-defined, the long-term health consequences of others—particularly at environmentally relevant concentrations—remain a critical area for future epidemiological investigation.

The total pollutant concentrations were lower in the effluent than in the influent samples, with removal rates for pesticides, other pharmaceuticals, esters, and other organic compounds were 93%, 34%, 58%, and 86%, respectively. While these results demonstrate that the wastewater treatment process effectively reduces the overall chemical load, the persistence of various pollutants in the effluent indicates a need for further optimization of current treatment protocols [34,35]. The ketone concentrations increased mainly because of benzophenone (the concentration of which increased by a factor of 40), and the concentrations of amines and derivatives increased mainly because of 2-aminophenol (the concentration of which increased by a factor of 23). It has been found that transformation products of pesticides and other compounds are often more toxic and difficult to degrade than the parent compounds [36]. Both pollutant removal efficiencies and the environmental risks posed by transformation products must therefore be assessed when developing wastewater treatment systems [37]. The formation of these intermediates can be hypothesized through specific oxidative pathways. Advanced oxidation processes (AOPs)—or analogous oxidative pathways in biological treatment—typically target the lone pair of electrons on nitrogen atoms, triggering N-dealkylation [38]. For example, in triazine-based pesticides, side-chain alkyl groups are preferentially eliminated, converting organic nitrogen into intermediate amides and amines, which are eventually mineralized into NH_4_^+^ and NO_3_^−^. Simultaneously, for aromatic amine pesticides, hydroxyl radicals (OH) or oxidative enzymes target the benzene ring to initiate hydroxylation, leading to ring cleavage [39]. Such non-selective bond cleavage frequently yields stable small-molecule intermediates, including aldehydes and ketones. Complex degradation sequences—encompassing ring-opening and molecular rearrangement—predominantly culminate in the formation of carbonyl groups (C=O), explaining the observed accumulation in ketone concentrations [40]. These findings highlight that while the treatment process reduces parent pesticide levels, it may lead to the accumulation of specific TPs, emphasizing the need for assessing the toxicity of the ‘whole effluent’ rather than just parent compounds.

Some pesticides can cause developmental toxicity in zebrafish larvae [41,42]. We found that the influent and effluent samples significantly negatively affected zebrafish larva survival and hatchability. Acute toxicity was found in the influent at high concentrations, but the effluent did not result in 100% mortality even at high concentrations. Developmental toxicity was stronger in the influent than effluent, and the toxic effects included increased heart rate, decreased body length, and a higher incidence of malformations (at much lower concentrations of the influent than effluent). This indicated that the wastewater treatment processes decreased the toxicity of the contaminant mixture to some extent.

In our assays, locomotor behavior emerged as the most sensitive phenotypic toxicity endpoint, consistent with previous findings in pesticide toxicity research [43]. Zhang et al. demonstrated that zebrafish behavioral changes were more sensitive than in vitro assays when evaluating antidepressants in wastewater. These results underscore that zebrafish behavior assays not only offer high sensitivity for environmental toxicity evaluation but also provide valuable insights into potential impacts on human health. Behavioral analyses indicated that the effluent caused abnormalities in locomotor behavior of zebrafish larvae, including increased lag time, decreased total travel distance, and altered locomotor speed, which may be related to pollutants interfering with the nervous system [44]. Alterations in early locomotor behavior of zebrafish larvae are often associated with neural development, so we used central and motor nerve transgenic zebrafish Tg (huc:eGFP) and Tg (hb9:eGFP) to assess the effects of the influent and effluent on neurodevelopment [41]. A previous study suggested that the pesticide emamectin benzoate adversely affected motor neuron axon length in Tg (hb9:eGFP) zebrafish and central nervous system neuron development in Tg (huC:eGFP) zebrafish and significantly inhibited locomotor behavior in zebrafish [12]. Consistent with this, we found that the effluent significantly decreased motor neuron axon length and the central nervous system fluorescence intensity, and that the effects were stronger for the influent than the effluent. This indicated that the pollutants in the influent and effluent may negatively affect the development of the zebrafish nervous system. The genes syn2a, gfap, and shha play key regulatory roles in neuronal development, transmitter release, and synapse formation [45]. Inflammation, oxidative stress, and neuronal apoptosis are key mechanisms leading to neurodevelopmental toxicity, and it has been found that exposure to various chemical pollutants can cause neurodevelopmental damage in zebrafish by causing neuronal apoptosis through inflammation and oxidative stress [46,47]. We found that exposure to the influent and effluent caused inflammation (Il-1β), oxidative stress (Cu/Zn-Sod and Mn-Sod), apoptosis (p53), and disturbed mRNA expression of key genes for neurodevelopment (Shha, Syn2a, and gfap), and that the influent was more toxic than the effluent. The results indicated that the effects of the influent and effluent on zebrafish locomotor behavior may have been related to changes in inflammation, oxidative stress, apoptosis, and expression of neurodevelopment-related genes. Stronger neurodevelopment and locomotor impairment occurred in the influent group than the effluent group, suggesting that the wastewater treatment processes decreased the total toxicity of the pollutants. The toxicity validation experiments demonstrated that the mixed exposure of BPMC, HEX, and MIPC at environmental concentrations exhibited toxic effects similar to those of the effluent sample, with additive or synergistic toxicity observed in the mixed exposure. In the process of chemical, screening, we only validated and quantified three significant pesticides, as they exhibited the highest risk rankings based on our initial assessment. But this strategy may not always accurately screen out other detected chemicals, such as 2-aminophenol and benzophenone. More precise identification methods of key toxicants are recommended in further studies. Specifically, the risk ranking in this study was primarily based on acute LC_50_ values because chronic toxicity data, such as NOEC, are currently limited for many detected contaminants and their transformation products. Consequently, while this ranking identifies primary acute toxicants, it may underestimate the potential for sublethal or chronic effects—such as behavioral alterations and neurodevelopmental impairments—which often occur at concentrations significantly lower than lethal thresholds. Consequently, future research will focus on filling these data gaps by incorporating targeted validation of key intermediates and chronic toxicity values to provide a more exhaustive assessment. Furthermore, the discrepancy between the two models arises because SSD curves are typically constructed based on extensive acute lethality data (LC_50_/EC_50_) or general growth inhibition data. If the database lacks highly sensitive neurotoxic endpoints (such as behavioral abnormalities) specific to these pesticides, the PNEC derived from the SSD model may be overestimated, resulting in an insufficiently conservative threshold. In contrast, AF method offers greater protection by applying a substantial safety factor (e.g., 100 or 1000) to the toxicity data of the most sensitive species. When specific toxicological endpoint data are limited, the AF method often provides a more robust margin of safety than the SSD model, thereby ensuring better protection against sensitive adverse effects like neurotoxicity. When specific toxicological endpoint data are limited, the AF method often provides a more robust margin of safety than the SSD model, thereby ensuring better protection against sensitive adverse effects like neurotoxicity.

## 5. Conclusions

This study provides direct evidence that pesticide manufacturing wastewater poses risks to aquatic ecosystems, with HEX, BPMC and MIPC identified as key toxicants exhibiting additive or synergistic effects. Zebrafish embryo assays revealed that locomotor behavior is a more sensitive endpoint, than traditional acute lethal or teratogenic measures. Although the wastewater treatment process effectively reduces the overall chemical load, in vivo zebrafish assays demonstrated that both influent and effluent samples, even at low concentrations, induced significant inflammation, oxidative stress, and apoptosis, leading to neurodevelopmental impairment in larvae. These findings underscore the limitations of conventional treatment in eliminating complex mixture toxicity. Furthermore, this study represents an integration of non-target chemical screening with the assessment of both apical and non-apical endpoints, enabling a comprehensive evaluation of pesticide wastewater toxicity. Future research should focus on optimizing wastewater treatment processes to eliminate recalcitrant toxicants and developing standardized behavioral assays for routine monitoring, ultimately mitigating the ecological and human health risks of pesticide wastewater.

## Figures and Tables

**Table 1:** Concentrations of contaminants determined by untargeted liquid chromatography high-resolution mass spectrometry.

Class	Mean Concentration (μg/L)	Mean Concentration (μg/L)	Removal Efficiency (%)
	Influent	Effluent	
ΣDrugs-Antibiotics	2.76	3.14	−14%
ΣDrugs-Pesticide	7.98	0.53	93%
ΣDrugs-Others	12.31	8.19	34%
ΣOrganic compounds-amines and derivatives	3.88	11.51	−197%
ΣOrganic compounds-Nitrogen compounds	4.12	7.42	−80%
ΣOrganic compounds-Ketones	2.87	27.72	−867%

**Table 2:** Risk ranking of detected concentrations and toxicity ratios of CECs in effluent.

Name	CAS	C (Effluent Concentration (μg/L))	96 h-LC_50_ (mg/L)	14d-NOEC(mg/L)	Ratio (C/LC_50_)	Ranking
Isoprocard	2631-40-5	0.337	22	/	0.00002	1
Hexaconazole	22778-07-2	2.076	12	/	0.0002	2
Fenobucarb	3766-81-2	3.225	1.7	0.2	0.002	3
Cyanophos	297-97-2	0.266	0.09	/	0.003	4
Thiobencarb	28249-77-6	4.414	0.98	0.11	0.005	5
Dithiopyr	97886-45-8	1.848	0.36	/	0.005	6
Cadusafos	95465-99-9	0.853	0.13	/	0.007	7
Allidochlor	93-71-0	25.078	2	/	0.013	8
Triethyl phosphate	78-40-0	1699.81	100	/	0.017	9
Triisobutyl phosphate	126-71-6	337.22	17.8	/	0.019	10
Naphthalen-2-amine	91-59-8	151.3	3.9	/	0.039	11
Carbamazepine	298-46-4	1034.96	19.9	/	0.052	12
Diethyl phthalate	84-66-2	1826.45	16.7	/	0.109	13
Genistein	446-72-0	360.36	1.9	/	0.190	14
3,4-Dichloroaniline	95-76-1	915.66	3.22	0.486	0.284	15

## Data Availability

The original contributions presented in this study are included in the article/Supplementary Material. Further inquiries can be directed to the corresponding authors.

## Supplementary Materials

The following supporting information can be downloaded at: https://www.mdpi.com/article/10.3390/biology15090700/s1, Figure S1: General mixture toxicity. (A) body length, (B) movement speed, and (C) movement distance; Table S1: The primer sequence of the gene; Table S2: Screening of chemicals in water samples from influent and effluent; Table S3: 1. Species effect values of Hexaconazole; 2. Species effect values of Fenobucarb; 3. Species effect values of Isoprocar; Table S4: SSD method for PNEC; Table S5: AF method for PNEC.
