# Supplementary material for: Multi-Dimensional Assessment Approach to Assess Pesticide Manufacturing Industry Wastewater Toxicity
Source: Biology (Basel). 2026 Apr 29;15(9):700. doi: 10.3390/biology15090700 (PMC13162825; doi:10.3390/biology15090700)
Supplement: Supplementary file 1 [file biology-15-00700-s001.zip › biology-4246754-supplementary.pdf]

Multi-dimensional assessment approach to assess pesticide manufacturing industry wastewater toxicity

Deling Fan[a,b], *Jian Wang*[b], *Lili Shi*[b], *Lei Wang*[b*], *Zheng Fang*[a*]

[a]School of Biological and Pharmaceutical Engineering, Nanjing University of Technology, Nanjing, 211816, China

[b]Nanjing Institute of Environmental Science, Ministry of Ecology and Environment, Nanjing, 210042, China

Address for Correspondence:

*Lei Wang**

Nanjing Institute of Environmental Science, Ministry of Ecology and Environment, Nanjing, 210042, China

Email: wanglei@nies.org

*Zheng Fang**

School of Biological and Pharmaceutical Engineering, Nanjing University of Technology, Nanjing, 211816, China

Email: fzcpu@njtech.edu.cn

*Corresponding authors

E-mails:wanglei@nies.org (Lei Wang). fzcpu@njtech.edu.cn (Zheng Fang)

Supplementary Materials

Table S1 The primer sequence of the gene.

Gene	Sequence
β-actin	Forward: 5′-GGTATGGGCCAGAAAGAC-3′
	Reverse: 5′-CTCCTCACGGGCTACTCT-3′
Cu/Zn-Sod	Forward: 5′-GTCGTCTGGCTTGTGGAGTG-3′
	Reverse: 5′-TGTCAGCGGGCTAGTGCTT-3′
syn2a	Forward: 5’-GTGACCATGCCAGCATTTC-3’
	Reverse: 5’-TGGTTCTCCACTTTCACCTT-3’
shha	Forward: 5’-TGTCCTCGACAACTCAACGG -3’
	Reverse: 5’- TCCGTGTATATCCGCTGCAC -3’
gfap	Forward: 5’- GGATGCAGCCAATCGTAAT-3’
	Reverse: 5’- TTCCAGGTCACAGGTCAG-3’
IL-1β	Forward: 5’- CATTTGCAGGCCGTCACA -3’
	Reverse: 5’- GGACATGCTGAAGCGCACTT -3’
P53	Forward: 5’-CCCGGATGGAGATAACTTG -3’
	Reverse: 5’-CACAGTTGTCCATTCAGCAC-3’
Mn-Sod	Forward: 5′-GTCCGCACTTCAACCCTCA-3′
	Reverse: 5′-TCCTCATTGCCACCCTTCC-3′

Table S2 Screening of chemicals in water samples from influent and effluent.

Chemical	CAS Number
**Drugs - Antibiotics**	
sulfacetamide	144-80-9
sulfamerazine	127-79-7
sulfisoxazol	127-69-5
sulfabenzamide	127-71-9
cinoxacin	28657-80-9

oxolinic acid	14698-29-4
nalidixic acid	389-08-2
flumequine	42835-25-6
Gatifloxacin	112811-59-3
Florfenicol	73231-34-2
4-Epianhydrotetracycline hydrochloride	4465-65-0
cefotaxime	63527-52-6
clindamycin hydrochloride	21462-35-9
**Drugs - Pesticide**	
prometryn	7287-19-6
Diethyltoluamide	134-62-3
Difenoxuron	14214-32-5
Fenobucarb	3766-81-2
Dithiopyr	97886-45-8
Cadusafos	95465-99-9
Cyanophos	297-97-2
Thiobencarb	28249-77-6
Isoprocard	2631-40-5
Hexaconazole	22778-07-2
Allidochlor	93-71-0
Daminozide	1596-84-5
**Drugs - Others**	
Sarafloxacin hydrochloride	91296-87-6
carbamazepine	298-46-4
Propyzamide	23950-58-5
primidone	125-33-7
methimazole	60-56-0
propylthiouracil	51-52-5
amoxapin	14028-44-5
aminoglutethimide	125-84-8
altretamine	645-05-6
ifosfamide	3778-73-2
Ormetoprim	6981-18-6
aspartame	22839-47-0
alachlor	15972-60-8
genistein	446-72-0
sulindac	38194-50-2
etodolac	41340-25-4
estrone	53-16-7
ibuprofen	15687-27-1
nifedipine	21829-25-4
atenolol	29122-68-7

mebendazole	31431-39-7
**Organic compounds - amines and derivatives**	
2-Aminophenol	95-55-6
m-Toluidine	108-44-1
o-Anisidine	90-04-0
2,6-Dimethylanilin	87-62-7
2-Methoxy-5-methylaniline	120-71-8
4-isopropylaniline	99-88-7
naphthalen-1-amine	134-32-7
naphthalen-2-amine	91-59-8
4-Chloro-2-methylanilin	95-69-2
4-Aminobiphenyl	92-67-1
Diphenylamine	122-39-4
3,4-dichloroaniline	95-76-1
4,4′methylenebis(2-chloroaniline)	101-14-4
**Organic compounds - Nitrogen compounds**	
N-Nitrosodipropylamine	621-64-7
1-Nitrosopyrrolidine	930-55-2
2-Amino-9H-pyrido[2,3-b]indole	26148-68-5
4-(methylnitrosamino)-1-(3-pyridyl)-1-butanone	64091-91-4
N-ethyl-N-nitroso-ethanamine	55-18-5
3,4-dimethylimidazo[4,5-f]quinolin-2-amine	77094-11-2
**Organic compounds - Ketones**	
Isophorone	78-59-1
Benzophenone	119-61-9
Michler's ketone	90-94-8
**Organic compounds - Esters**	
Trimethyl phosphate	512-56-1
Triethyl phosphate	78-40-0
Tripropyl phosphate	513-08-6
Bis(2-methoxyethyl) phthalate	117-82-8
Diethyl phthalate	84-66-2
tris(1-chloropropan-2-yl) phosphate	13674-84-5
Tris(2-methylpropyl) phosphate	126-71-6
Dibutyl phosphate	107-66-4
2-Ethylhexyl diphenyl phosphate	1241-94-7
**Organic compounds - Others**	
Carbanilic acid	04243-04-3
Titanium Dioxide	9342-3-4

Sudan I	842-07-9
cotinine	486-56-6
N-methylacetamide	79-16-3
imidazolidine-2-thione	96-45-7

Table S3-1 Species effect values of Hexaconazole

Phylum	Family	Genus	Species	LC_{50}(mg/L)	day
cyanophyta	*Chroococcaceae*	*Microcystis*	*aeruginosa*	4.061	4
chlorophyta				2.284	4
	Pleurochloridaceae	*Scenedesmus*	*acutus*	0.866	4
				1.85	4
				1.025	4
Chlorella	*chlorella*	*Chlorella*	*vulgaris*	2.893	4
				0.5	4
	Chlorophyta	*Dunaliella*	*tertiolecta*	0.91	2
	Dunalieceae	*Anabaena*	*flosaquae*	7.2	4
Arthropoda				4	4
				3.5	4
				1.9	4
	Daphnia	*Daphnia*	*magna*	1.6	4
				10	4
				4.7	4
Vertebrata		*Danio*	*rerio*	5.97	4
		Lepomis	*macrochirus*	5.1	4
	Cyprinidae			3.8	4
		Oncorhynchus	*mykiss*	6.7	4

Table S3-2 Species effect values of Fenobucarb

Phylum	Family	Genus	Species	LC_{50}(mg/L)	day

Chlorophyta	*Selenastraceae*	*Raphidocelis*	*subcapitata*	33	3
Vertebrata	*Ranidae*	*Rana*	*limnocharis*	8.648	2
arthropod	*Astacidae*	*Paratya*	*compressa*	0.00505	4
Arthropoda	*Daphnia*	*Daphnia*	*magna*	0.035	2
Vertebrata	*cichlidae*	*niloticus*	*niloticus*	3.6	4
				1.47	4
	Cyprinidae	*Carassius*	*auratus*	25.2	4
				5.4	4
	Cichlid	*Oreochromis*	*niloticus*	6.12	4
				3.6	4
				0.1716	4
	cyprinidae	*Cyprinus*	*carpio*	1.7	4
				5.8	4

Table S3-3 Species effect values of Isoprocar

Phylum	Family	Genus	Species	LC_{50}(mg/L)	day
Chlorella	*Chlorella*	*Chlorella*	*vulgaris*	24.05	4
				2.1	4
	Scenedesmus	*acutus*	*acutus*	5.02	4
				3.79	4
	Chroococcaceae	*Microcystis*	*aeruginosa*	26.69	4
				7.2	4
Cyanophyta	*Nostocaceae*	*Anabaena*	*flosaquae*	66.65	4
Chordata	*Poeciliidae*	*Gambusia*	*affinis*	12	2
		Poecilia	*reticulata*	1.6	2
	Cyprinidae	*Cyprinus*	*carpio*	5.3	4
				32	4
		Cyprinus	*carpio*	1	21
		Cyprinidae	*carpio*	4.61	4

Physella	*acuta*	6.5	2

Table S4 SSD method for PNEC

	TEF	$PNEC_{SSD}$	$C_{effluent}$ (ug/L)	10%$C_{effluent}$ (ug/L)
MIPC	0.0313	0.446 mg/L	0.337	0.0337
BPMC	1	0.014 mg/L	3.225	0.3225
HEX	0.076	0.185 mg/L	2.076	0.2076
$C_{effluent,BPMC} \times 1 + C_{effluent,MIPC} \times 0.0313 + C_{effluent,HEX} \times 0.076 \leq 14 \times 36.1 \times 2 \times DF_2$				

Table S5 AF method for PNEC

	TEF	$PNEC_{AF}$	$C_{effluent}$ (ug/L)	10%$C_{effluent}$ (ug/L)
MIPC	0.0035	10 μg/L	0.337	0.0337
BPMC	1	0.035μg/L	3.225	0.3225
HEX	0.07	0.5μg/L	2.076	0.2076
$C_{effluent,BPMC} \times 1 + C_{effluent,MIPC} \times 0.0035 + C_{effluent,HEX} \times 0.07 \leq 0.035 \times 36.1 \times 2 \times DF_2$				

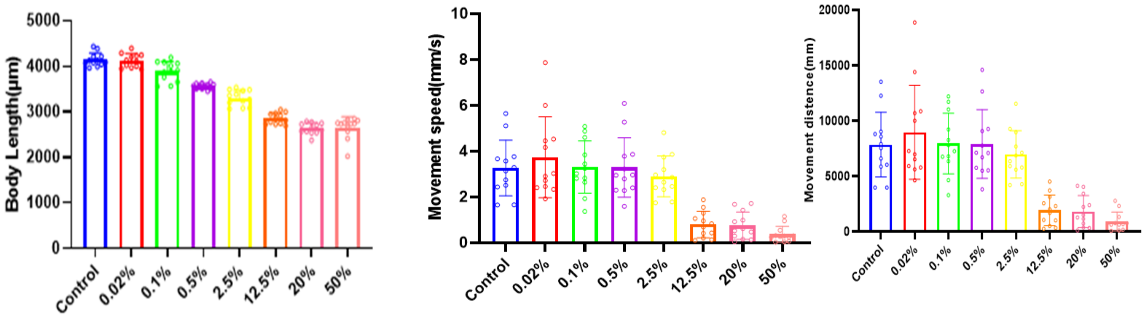

(A) (B) (C)

Figure S1. General mixture toxicity. (A) body length , (B) movement speed, and (C) movement distance.
